# Autophagy Protects against Palmitic Acid-Induced Apoptosis in Podocytes in vitro

**DOI:** 10.1038/srep42764

**Published:** 2017-02-22

**Authors:** Xu-shun Jiang, Xue-mei Chen, Jiang-min Wan, Hai-bo Gui, Xiong-zhong Ruan, Xiao-gang Du

**Affiliations:** 1 Department of Nephrology, The First Affiliated Hospital of Chongqing Medical University, Youyi Road 1, Chongqing, 400042, China; 2 Emergency Department, The First Affiliated Hospital of Chongqing Medical University, Youyi Road 1, Chongqing, 400042, China; 3 Centre for Nephrology, Royal Free and University College Medical School, University College London, Royal Free Campus, Rowland Hill Street, London, NW3 2PF, United Kingdom; 4 Centre for Lipid Research, Key Laboratory of Molecular Biology on Infectious Diseases, Ministry of Education, Chongqing Medical University, Youyi Road 1, Chongqing, 400042, China; 5 Laboratory of Lipid & Glucose Metabolism, The First Affiliated Hospital of Chongqing Medical University, Youyi Road 1, Chongqing, 400042, China

## Abstract

Autophagy is a highly conserved degradation process that is involved in the clearance of proteins and damaged organelles to maintain intracellular homeostasis and cell integrity. Type 2 diabetes is often accompanied by dyslipidemia with elevated levels of free fatty acids (FFAs). Podocytes, as an important component of the filtration barrier, are susceptible to lipid disorders. The loss of podocytes causes proteinuria, which is involved in the pathogenesis of diabetic nephropathy. In the present study, we demonstrated that palmitic acid (PA) promoted autophagy in podocytes. We further found that PA increased the production of reactive oxygen species (ROS) in podocytes and that NAC (N-acetyl-cysteine), a potent antioxidant, significantly eliminated the excessive ROS and suppressed autophagy, indicating that the increased generation of ROS was associated with the palmitic acid-induced autophagy in podocytes. Moreover, we also found that PA stimulation decreased the mitochondrial membrane potential in podocytes and induced podocyte apoptosis, while the inhibition of autophagy by chloroquine (CQ) enhanced palmitic acid-induced apoptosis accompanied by increased ROS generation, and the stimulation of autophagy by rapamycin (Rap) remarkably suppressed palmitic acid-induced ROS generation and apoptosis. Taken together, these *in vitro* findings suggest that PA-induced autophagy in podocytes is mediated by ROS production and that autophagy plays a protective role against PA-induced podocyte apoptosis.

Diabetic nephropathy (DN), the primary cause of end-stage renal disease (ESRD), has become a serious health problem in many developing and developed countries. Proteinuria is the primary clinical manifestation of diabetic nephropathy, and it is also an independent risk factor for the progression of diabetic nephropathy^1^. In diabetic nephropathy, proteinuria is mainly caused by the disruption of the structure and function of the glomerular filtration membrane^2^. Diabetes mellitus is often associated with disorders of lipid metabolism, characterized by hypertriglyceridemia, which is closely related to the occurrence and development of diabetic nephropathy and even the progression to end-stage renal disease^3^,^4^. However, the exact mechanism of how lipid disorders accelerate the progress of diabetic nephropathy, in particular the role of hypertriglyceridemia in podocyte injury, is still uncertain.

Podocytes play a crucial role in maintaining the glomerular filtration barrier^5^,^6^. Injury, apoptosis and detachment of podocytes lead to proteinuria in patients with diabetic nephropathy, and the reduction in the number of podocytes is an important index used to predict the progression of DN^7^,^8^. More importantly, podocytes are highly specialized, terminally differentiated visceral epithelial cells with a very limited ability for cell regeneration. Therefore, maintaining the normal structure and function of podocytes, and protecting podocytes from injury and apoptosis have become a major target for the treatment of diabetic nephropathy.

The degradation of intracellular proteins occurs mainly through the ubiquitin-proteasome system (UPS) and autophagy pathways. Autophagy is a highly conserved lysosomal degradation pathway that degrades cellular unfolded/misfolded proteins and damaged/unnecessary organelles, to maintain intracellular homeostasis and cell survival. Three main types of autophagy have been recognized in cells, including macroautophagy, microautophagy, and chaperone-mediated autophagy^9^. Accumulating evidence indicates that autophagy is closely related to the pathogenesis of many important kidney diseases such as acute kidney injury (AKI)^10^,^11^,^12^, polycystic kidney disease (PKD)^13^,^14^, focal segmental glomerular sclerosis (FSGS)^15^, and diabetic nephropathy (DN)^16^, among others. Additionally, a strong body of evidence suggests that autophagy plays a critical role in kidney maintenance and diseases^17^ and that insufficient podocyte autophagy may be involved in the pathogenesis of podocyte loss, leading to massive proteinuria and the rapid progression of diabetic nephropathy, and autophagy is therefore considered to be a potential novel therapeutic strategy for DN^18^,^19^,^20^. Podocytes are susceptible to saturated FFAs^21^, and it has been reported that high-fat feeding enhanced autophagy in beta cells^22^ and that palmitic acid (PA) induced autophagy in hepatocytes^23^. However, the exact role of autophagy in the podocytes of diabetic nephropathy patients with dyslipidemia is still not fully elucidated.

In this study, we aimed to investigate the significance of autophagy in palmitic acid-induced podocyte apoptosis using an *in vitro* model and to clarify its mechanism.

## Results

### Palmitic acid induced autophagy in podocytes

**Figure 1:**
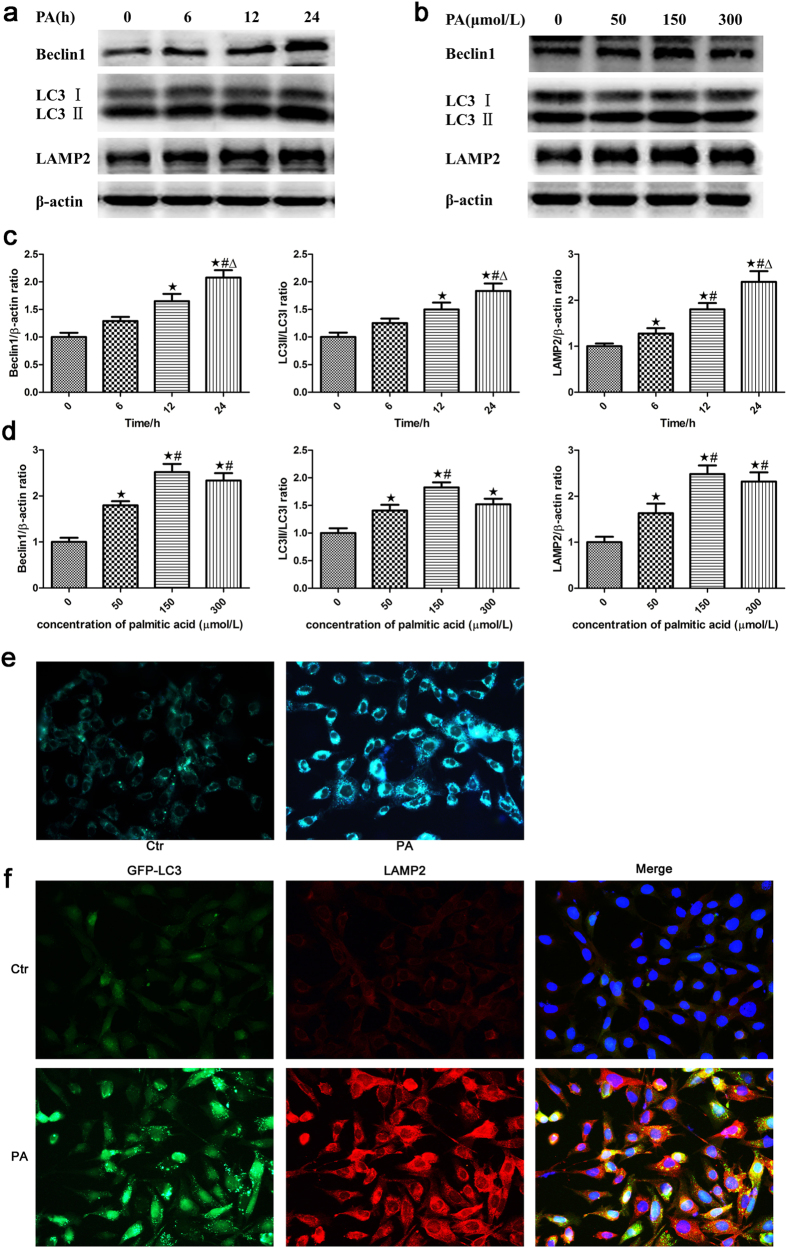
Palmitic acid induced autophagy in podocytes. (**a**) Representative Western blot analyses of Beclin1, LC3-II/LC3-I and LAMP-2 expression in podocytes treated with 150 μmol/L palmitic acid at different time points. (**b**) Representative Western blot analyses of Beclin1, LC3-II/LC3-I and LAMP-2 expression in podocytes treated with different concentrations of palmitic acid for 24 hours. (**c**,**d**) Densitometric analysis of Beclin1, LC3-II/LC3-I and LAMP-2 expression in Figure a and b, respectively. The data are represented as the mean ± SEM from at least 3 independent experiments. ∗p < 0.05 vs. 0 h, ^#^p < 0.05 vs. 6 h, Δ p < 0.05 vs. 12 h (**c**). ∗p < 0.05 vs. 0 μmol/L, ^#^p < 0.05 vs. 50 μmol/L (d). (**e**) Podocytes were treated with 150 μmol/L palmitic acid for 24 hours and then incubated with monodansylcadaverine (MDC) for 15 min. The cells were washed and examined under a fluorescence microscope (400×). (**f**) Podocytes stably expressing GFP-LC3 were treated with 150 μmol/L palmitic acid for 24 hours and then stained with an antibody against LAMP-2. Representative images of the cells were taken with a fluorescence microscope (400×). Ctr: Control group, podocytes were treated with 1% bovine serum albumin (BSA); PA: Palmitic acid group, podocytes were treated with 150 μmol/L palmitic acid for 24 hours.

To examine the effect of lipid disorders on autophagy in podocytes, we treated MPC5 cells with 150 μmol/L PA, a saturated FFA, for different lengths of time and found that the expression of Beclin1, LC3-II/LC3-I and LAMP-2 in podocytes increased in a time-dependent manner, as demonstrated by western blot analysis (Fig. 1a and c). In addition, a dose-dependent increase in Beclin1, LC3-II/LC3-I and LAMP-2 expression was observed in podocytes after exposure to palmitic acid for 24 hours (Fig. 1b and d). These results demonstrated that high lipid levels increased the expression of autophagy-related proteins in podocytes. However, there was no significant difference in Beclin1, LC3-II/LC3-I and LAMP-2 expression found between podocytes treated with 150 μmol/L and 300 μmol/L palmitic acid for 24 hours (Fig. 1b and d), which suggested that 150 μmol/L was the appropriate dose of palmitic acid for the induction of autophagy.

Autophagosomes are autophagy vesicles with an acidic double-membrane structure, which can be detected by the autofluorescent agent MDC. Treatment of podocytes with 150 μmol/L PA resulted in a clear increase in fluorescence intensity and the number of autophagic vacuoles in PA-induced podocytes compared to the control cells (Fig. 1e).

As LC3 is a major constituent of the autophagosome, we established a stable GFP-LC3- expressing MPC5 cell line to visualize autophagosome formation in real time in live cells and observed a diffuse cytoplasmic localization of GFP-LC3 in the control group, whereas the PA-treated cells exhibited increased punctate fluorescence (Fig. 1f), suggesting that cytoplasmic LC3 is processed and recruited to autophagosomes. Lysosomal-associated membrane protein 2 (LAMP-2), an important constituent of the lysosomal membrane, is thought to be critical for the fusion of autophagosomes with lysosomes and subsequent lysosomal degradation^24^. In our study, we also found that PA increased LAMP-2 expression and observed the colocalization of LAMP-2 with LC3 in podocytes (Fig. 1f), indicating that PA increased autolysosome formation and the fusion of autophagosomes with lysosomes in podocytes.

### The effect of palmitic acid on autophagic flux in podocytes

**Figure 2:**
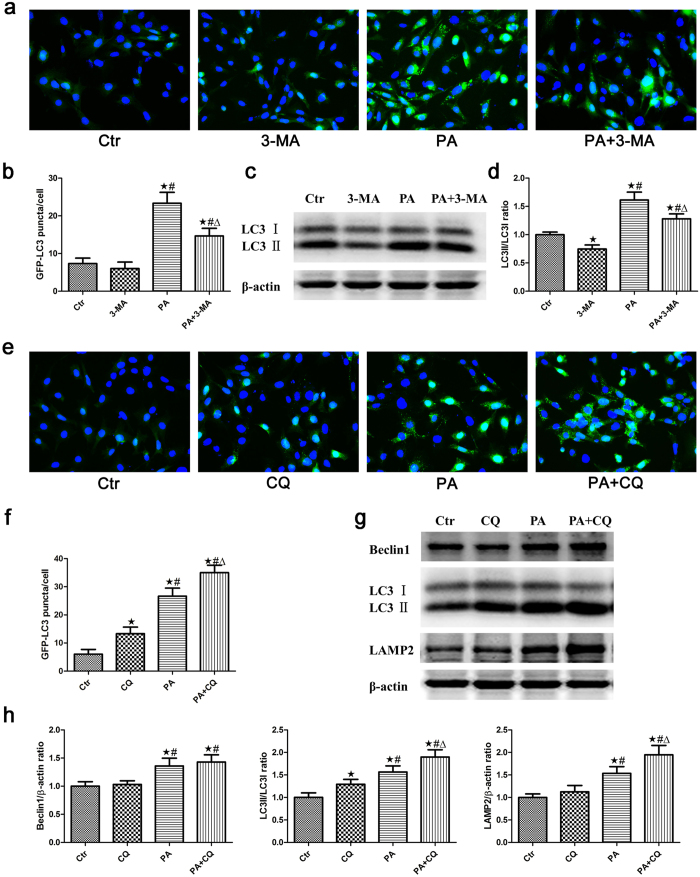
The effect of palmitic acid on autophagic flux in podocytes. (**a**) Podocytes stably expressing GFP-LC3 were pretreated with 2 mmol/L 3-MA for 1 hour, treated with 150 μmol/L palmitic acid for 24 hours and then analyzed by fluorescence microscopy (400×). (**b**) GFP-LC3 puncta (mean ± SEM) were quantified for each experiment (n = 3). At least 30 cells were counted in each individual experiment. ∗p < 0.05 vs. control group, ^#^p < 0.05 vs. 3-MA group, Δp < 0.05 vs. PA group. (**c**) Podocytes were treated with 150 μmol/L palmitic acid in the absence or presence of 2 mmol/L 3-MA for 24 hours, and the cell lysates were then analyzed by immunoblot using an antibody against LC3. (**d**) Densitometric analysis of LC3-II/LC3-I expression in Figure c. ∗p < 0.05 vs. control group, #p < 0.05 vs. 3-MA group, Δp < 0.05 vs. PA group. (**e**) Podocytes stably expressing GFP-LC3 were pretreated with 3 μmol/L CQ for 1 hour, treated with 150 μmol/L palmitic acid for 24 hours and then analyzed by fluorescence microscopy (400×). (**f**) GFP-LC3 puncta fluorescence (mean ± SEM) were quantified for each experiment (n = 3). At least 30 cells were counted in each individual experiment. ∗p < 0.05 vs. control group, #p < 0.05 vs. CQ group, Δp < 0.05 vs. PA group. (**g**) Podocytes were treated with 150 μmol/L palmitic acid in the absence or presence of 3 μmol/L CQ for 24 hours, and the cell lysates were then analyzed by immunoblot using antibodies against Beclin1, LC3 and LAMP-2. (**h**) Densitometric analysis of Beclin1, LC3-II/LC3-I and LAMP-2 expression in Figure g. ∗p < 0.05 vs. control group, ^#^p < 0.05 vs. CQ group, Δp < 0.05 vs. PA group. Ctr: Control group, podocytes were treated with 1% BSA; 3-MA: Podocytes were treated with 2 mmol/L 3-MA for 24 hours; PA: Palmitic acid group, podocytes were treated with 150 μmol/L palmitic acid for 24 hours; PA + 3-MA: Podocytes were treated with 150 μmol/L palmitic acid for 24 hours after pretreatment with 2 mmol/L 3-MA for 1 hour; CQ: Podocytes were treated with 3 μmol/L CQ for 24 hours; PA + CQ: Podocytes were treated with 150 μmol/L palmitic acid for 24 hours after pretreatment with 3 μmol/L CQ for 1 hour.

As the increase in autophagosome and autolysosome formation could result from increased autophagic activity and decreased lysosomal activity, we further used autophagy inhibitors to investigate the effect of PA on autophagic flux in podocytes. 3-Methyladenine (3-MA), an inhibitor of phosphatidylinositol 3-kinase class III (PI3KC3), can inhibit the formation of Beclin1-PI3KC3 complexes, the conversion of soluble LC3-I to lipid bound LC3-II and the formation of autophagosomes^25^. In the present study, we found that 3-MA suppressed the PA-induced formation of GFP-LC3 puncta (Fig. 2a), and the effect was further confirmed by a western blot analysis of LC3 expression (Fig. 2c), indicating that PA induced the formation of autophagosomes in podocytes. Our results further showed that chloroquine (CQ), a lysosomal inhibitor^26^, significantly increased the accumulation of GFP-LC3-positive autophagosomes (Fig. 2e), LC3-II/LC3-I expression and LAMP-2 expression in PA-induced podocytes compared with the PA group. However, there was no statistically significant difference in Beclin1 expression in the PA + CQ group compared with the PA group (Fig. 2g and h). We also found that PA further enhanced GFP-LC3 puncta fluorescence in podocytes when autophagosomal degradation was blocked by treatment with chloroquine (Fig. 2e). Western blot analyses showed that PA significantly augmented the expression of Beclin1, LC3-II/LC3-I and LAMP-2 in podocytes in the presence of CQ compared with the CQ group (Fig. 2g). These findings suggested that PA increased autophagosome and autolysosome formation in podocytes.

### Role of oxidative stress in palmitic acid-induced autophagy in podocytes

**Figure 3:**
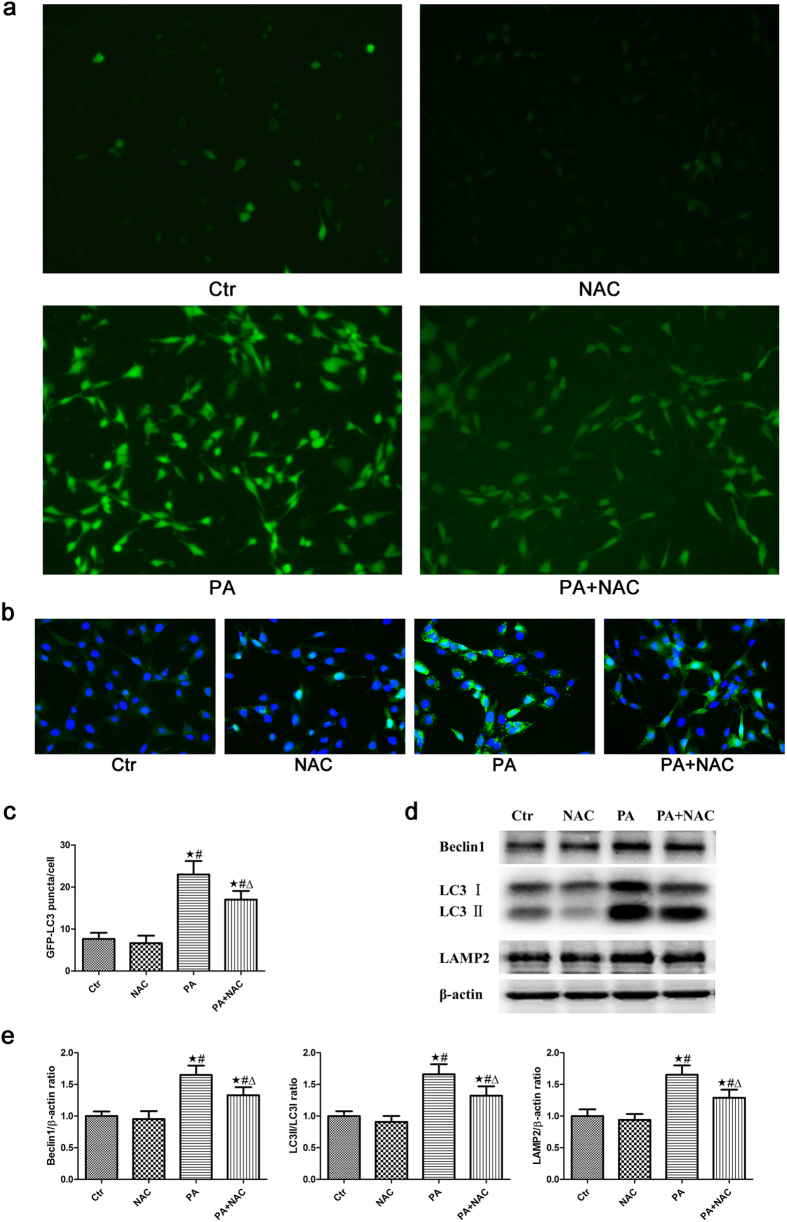
Role of oxidative stress in palmitic acid-induced autophagy in podocytes. (**a**) Representative immunofluorescence images of ROS in podocytes (200×). Podocytes were treated with 150 μmol/L palmitic acid for 24 hours with or without pretreatment of NAC (150 μmol/L), and the intracellular ROS level was then measured using the fluorescent probe 2′,7′-dichlorfluorescein-diacetate (DCFH-DA). (**b**) Podocytes stably expressing GFP-LC3 were pretreated with 150 μmol/L NAC for 2 hours, treated with 150 μmol/L palmitic acid for 24 hours and then analyzed by fluorescence microscope (400×). (**c**) GFP-LC3 puncta (mean ± SEM) were quantified for each experiment (n = 3). At least 30 cells were counted in each individual experiment.∗p < 0.05 vs. control group, #p < 0.05 vs. NAC group, Δp < 0.05 vs. PA group. (**d**) Podocytes were treated with 150 μmol/L palmitic acid in the absence or presence of 150 μmol/L NAC for 24 hours, and the cell lysates were then analyzed by immunoblot using antibodies against Beclin1, LC3 and LAMP-2. (**e**) Densitometric analysis of Beclin1, LC3-II/LC3-I and LAMP-2 expression in Figure d. ∗p < 0.05 vs. control group, ^#^p < 0.05 vs. NAC group, Δp < 0.05 vs. PA group. Ctr: Control group, podocytes were treated with 1% BSA. NAC: Podocytes were treated with 150 μmol/L N-acetyl-cysteine (NAC) for 24 hours. PA: Palmitic acid group, podocytes were treated with 150 μmol/L palmitic acid for 24 hours. PA + NAC: Podocytes were treated with 150 μmol/L palmitic acid for 24 hours after pretreatment with 150 μmol/L N-acetyl-cysteine (NAC) for 2 hours.

Using the reactive oxygen species (ROS)-sensitive fluorescent probe DCFH-DA to monitor cellular oxidative stress, we found that PA considerably increased ROS production in podocytes, and that the antioxidant reagent N-acetyl-cysteine (NAC) significantly attenuated the PA-induced increase in ROS production (Fig. 3a).

To determine whether oxidative stress is involved in PA- induced autophagy in podocytes, we treated podocytes with either bovine serum albumin (BSA) or 150 μmol/L PA in the presence or absence of 150 μmol/L NAC for 24 hours. The preincubation of podocytes with NAC dramatically blocked the PA-induced increase in GFP-LC3 puncta fluorescence (Fig. 3b). In addition, Western blot analyses showed that the PA-induced expression of autophagy-related proteins, including Beclin1, LC3-II/LC3-I and LAMP-2, also decreased in the presence of NAC (Fig. 3c). These findings suggested that oxidative stress plays a role in PA-induced autophagy.

### Palmitic acid-induced lipotoxicity promoted mitochondrial injury in podocytes

**Figure 4:**
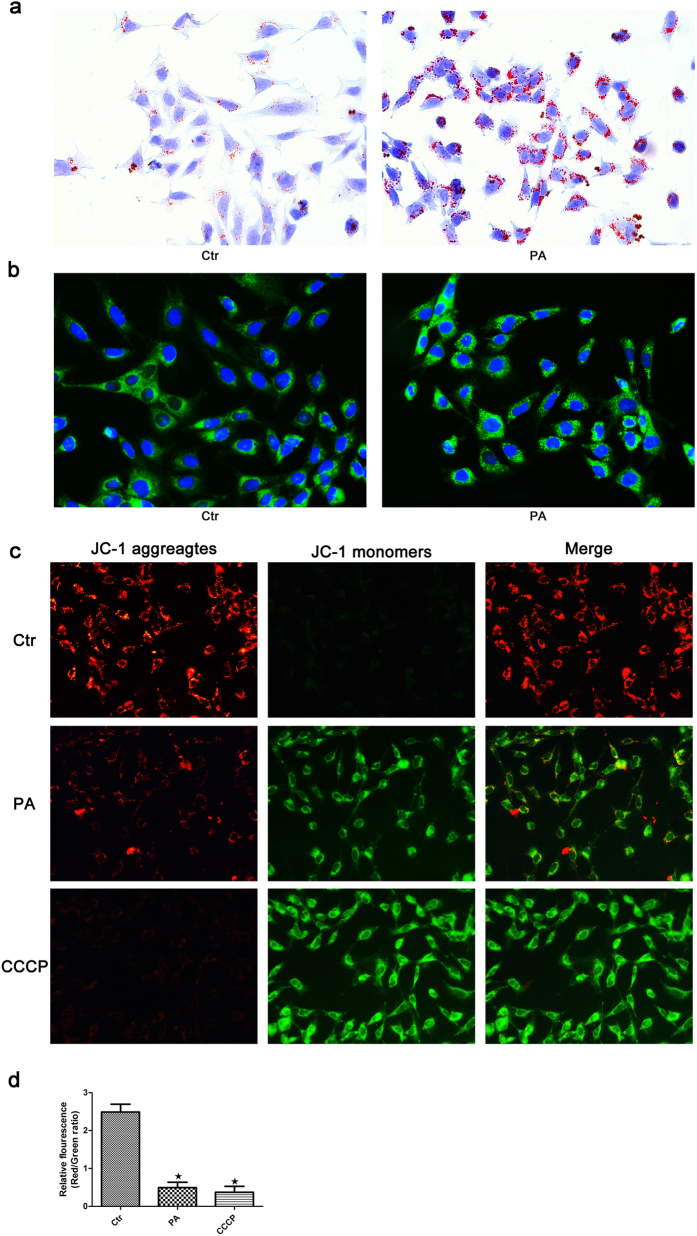
Palmitic acid-induced lipotoxicity promotes mitochondrial injury in podocytes. Podocytes were treated with 150 μmol/L palmitic acid for 24 hours, and the lipid content in the cells was then detected by Oil Red O staining (400×) (**a**) and BODIPY lipid probes (400×) (**b**). (**c**) Palmitic acid induced a change in mitochondrial transmembrane potential (ΔΨm). Podocytes were treated with 150 μmol/L palmitic acid for 24 hours or 10 μM CCCP for 1 hour, and the cells were then stained with the fluorescent probe JC-1. CCCP, a powerful mitochondrial uncoupling agent, was used as a positive control. Red fluorescence indicates normal ΔΨm with JC-1 aggregates in mitochondria, and green reflects cytosolic JC-1 monomer indicative of ΔΨm loss (400×). (**d**) Fluorescent intensity from 5 randomly selected microscopic fields per group was captured and analyzed. The data are expressed as the mean ± SEM; n = 3. ∗p < 0.05 vs. control group. Ctr: Control group, podocytes were treated with 1% BSA. PA: Palmitic acid group, podocytes were treated with 150 μmol/L palmitic acid for 24 hours. CCCP: Podocytes were treated with 10 μM CCCP for 1 hour.

Using Oil Red O staining and BODIPY lipid probes, we found that PA induced an increase in lipid accumulation in podocytes (Fig. 4a and b). As the cytotoxic accumulation of lipids in non-adipose tissues can lead to cellular lipotoxicity, we further investigated the effect of palmitate on mitochondrial injury in podocytes. Mitochondrial damage is often accompanied by a decrease in mitochondrial membrane potential (ΔΨm). We used the fluorescent probe JC-1 to monitor the changes in ΔΨm in podocytes and found a great deal of J-aggregates, which fluoresced red, with a small amount of J-monomers, which fluoresced green, in the control group, while more diffuse J-monomer fluorescence and less J-aggregate staining was observed in the PA group and in cells treated with the mitochondrial membrane potential disrupter CCCP (Fig. 4c). A fluorescence intensity analysis showed a significant decrease in the red/green fluorescence intensity ratio in the PA group and CCCP group compared to the control group (Fig. 4d), indicating that PA caused a reduction in ΔΨm and mitochondrial damage in podocytes.

### Palmitic acid induced apoptosis in podocytes

**Figure 5:**
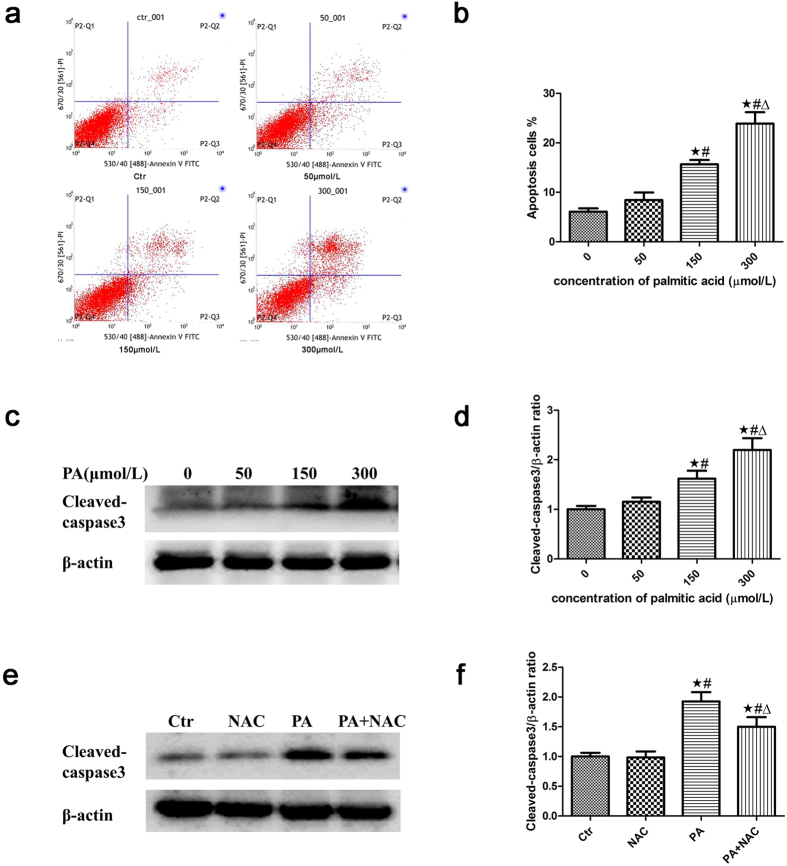
Palmitic acid induced apoptosis in podocytes. Representative images of flow cytometry analysis of podocytes after treatment with different concentrations of palmitic acid for 24 hours. (**a**) Representative cytograms. (**b**) Percentage of apoptotic cells. ∗p < 0.05 vs. 0 μmol/L, #p < 0.05 vs. 50 μmol/L, Δp < 0.05 vs. 150 μmol/L. (**c**) Representative Western blot analyses of cleaved-caspase3 expression in podocytes treated with different concentrations of palmitic acid for 24 hours. (**d**) Densitometric analysis of cleaved-caspase3 expression in Figure c. ∗p < 0.05 vs. 0 μmol/L, ^#^p < 0.05 vs. 50 μmol/L, Δp < 0.05 vs. 150 μmol/L. (**e**) Representative Western blot analyses of cleaved-caspase3 expression in podocytes treated with 150 μmol/L palmitic acid in the absence or presence of 150 μmol/L NAC for 24 hours. The cell lysates were analyzed by immunoblot using an antibody against cleaved-caspase3. (**f**) Densitometric analysis of cleaved-caspase3 expression in Figure e. The data are expressed as the mean ± SEM; n = 3. ∗p < 0.05 vs. control group, ^#^p < 0.05 vs. NAC group, Δp < 0.05 vs. PA group. Ctr: Control group, podocytes were treated with 1% BSA. NAC: Podocytes were treated with 150 μmol/L N-acetyl-cysteine (NAC) for 24 hours. PA: Palmitic acid group, podocytes were treated with 150 μmol/L palmitic acid for 24 hours. PA + NAC: Podocytes were treated with 150 μmol/L palmitic acid for 24 hours after pretreatment with 150 μmol/L N-acetyl-cysteine (NAC) for 2 hours.

We previously demonstrated that palmitic acid increased podocyte apoptosis and ROS production and that the antioxidant tempol significantly attenuated the enhanced ROS production and apoptosis^27^. In the present study, we confirmed that PA induced a dose-dependent increase in podocyte apoptosis using a flow cytometry assay (Fig. 5a and b) and by analyzing the expression of cleaved-caspase3, the activated form of caspase3 that is believed to play a critical role in the regulation of cell apoptosis^28^, by western blot (Fig. 5c and d). In addition, PA noticeably increased ROS production in podocytes (Fig. 3a), and the antioxidant reagent NAC ameliorated the increased apoptosis (Fig. 5e and f).

### Inhibition of autophagy increased PA-induced ROS generation and apoptosis in podocytes

**Figure 6:**
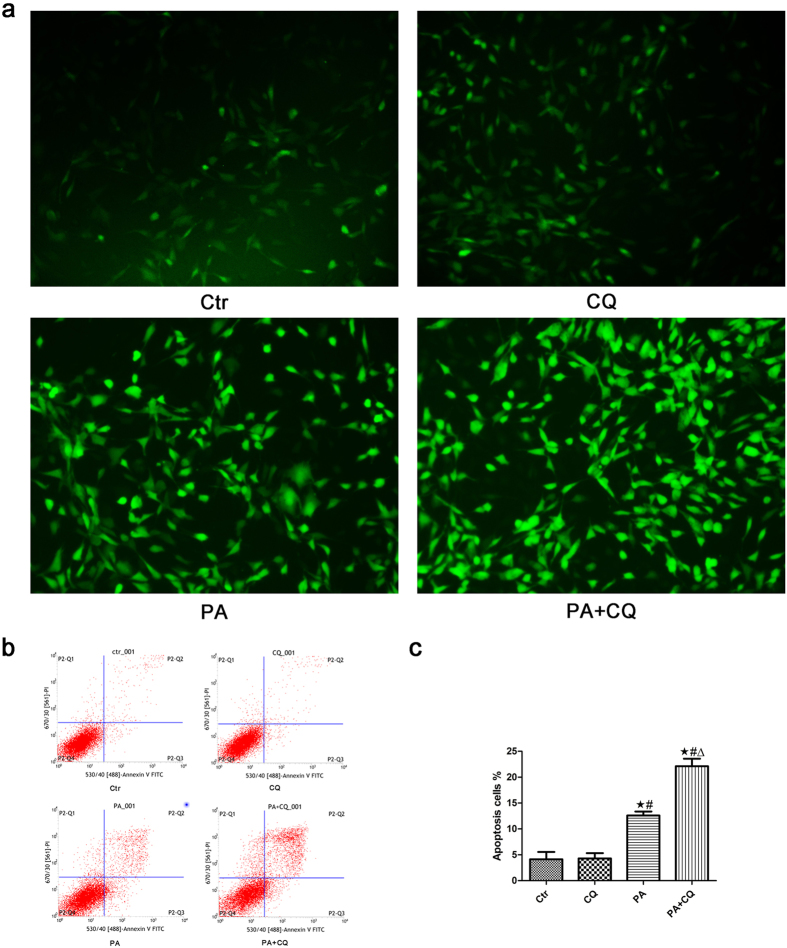
Inhibition of autophagy increased PA-induced ROS generation and apoptosis in podocytes. (**a**) Representative immunofluorescence images of ROS in podocytes (200×). Podocytes were treated with 150 μmol/L palmitic acid in the absence or presence of 3 μmol/L chloroquine (CQ) for 24 hours, and the intracellular ROS level was then measured using the fluorescent probe 2′,7′-dichlorfluorescein-diacetate (DCFH-DA). (**b,c**) Podocytes were treated with 150 μmol/L palmitic acid in the absence or presence of 3 μmol/L CQ for 24 hours, and apoptosis was measured using flow cytometry analysis. (**b**) Representative cytograms. (**c**) Percentage of apoptotic cells. ∗p < 0.05 vs. control group, ^#^p < 0.05 vs. CQ group, Δp < 0.05 vs. PA group. Ctr: Control group, podocytes were treated with 1% BSA. CQ: Podocytes were treated with 3 μmol/L chloroquine for 24 hours. PA: Palmitic acid group, podocytes were treated with 150 μmol/L palmitic acid for 24 hours. PA + CQ: Podocytes were treated with 150 μmol/L of palmitic acid for 24 hours after pretreatment with 3 μmol/L chloroquine for 1 hour.

To further determine the effect of autophagy on PA-induced ROS production and apoptosis, we treated podocytes with PA in the absence or presence of 3 μmol/L CQ for 24 hours and found that the suppression of autophagy by CQ further enhanced PA-induced ROS production (Fig. 6a) and apoptosis (Fig. 6b and c).

### Activation of autophagy suppressed PA-induced ROS generation and apoptosis in podocytes

**Figure 7:**
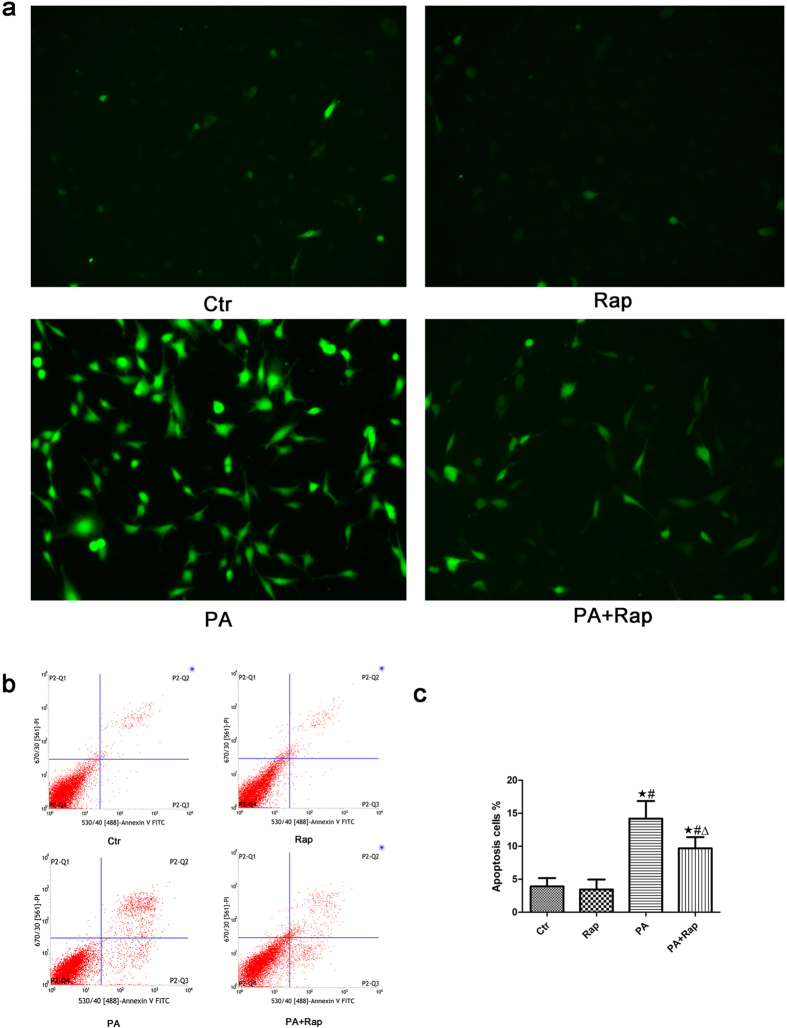
Activation of autophagy suppressed PA-induced ROS generation and apoptosis in podocytes. (**a**) Representative immunofluorescence images of ROS in podocytes (200×). Podocytes were treated with 150 μmol/L palmitic acid in the absence or presence of 1 ng/mL rapamycin (Rap) for 24 hours, and the intracellular ROS level was then measured using the fluorescent probe 2′,7′-dichlorfluorescein-diacetate (DCFH-DA). (**b,c**) Podocytes were treated with 150 μmol/L palmitic acid in the absence or presence of 1 ng/mL rapamycin for 24 hours, and apoptosis was measured using flow cytometry analysis. (**b**) Representative cytograms. (**c**) Percentage of apoptotic cells. ∗p < 0.05 vs. control group, ^#^p < 0.05 vs. Rap group, Δp < 0.05 vs. PA group. Ctr: Control group, podocytes were treated with 1% BSA. Rap: Podocytes were treated with 1 ng/mL rapamycin for 24 hours. PA: Palmitic acid group, podocytes were treated with 150 μmol/L palmitic acid for 24 hours. PA + Rap: Podocytes were treated with 150 μmol/L of palmitic acid for 24 hours after pretreatment with 1 ng/mL rapamycin for 1 hour.

Rapamycin (Rap) has been reported to induce autophagy in a variety of cell types, including podocytes^29^,^30^. We incubated podocytes with PA in the absence or presence of 1 ng/mL rapamycin for 24 hours and found that PA-induced ROS production (Fig. 7a) and apoptosis (Fig. 7b and c) were suppressed by treating podocytes with rapamycin, which suggested that the activation of autophagy protected against PA-induced podocyte apoptosis.

## Discussion

Autophagy is an essential and evolutionarily conserved intracellular degradation pathway involved in the degradation of long-lived proteins and cell organelles, to maintain the homeostasis of cell function and metabolism. Autophagic degradation is a tightly regulated and highly inducible process during which damaged proteins or organelles are encapsulated by the double-membrane structure of autophagy vesicles, which then fuse with lysosomes for degradation and recycling^9^. High levels of constitutive autophagy have been demonstrated in podocytes under basal conditions, and podocyte-specific deletion of autophagy-related 5 (Atg5) resulted in podocyte loss and late-onset glomerulosclerosis in aging mice^31^, highlighting the importance of autophagy in the maintenance of the function and integrity of podocytes. However, in certain settings^32^,^33^, uncontrolled massive autophagy may lead to cell death. It is still unclear how autophagy varies under lipid stress.

In this study, we found that PA increased the formation of autophagic vacuoles and the expression of autophagy-related proteins, including Beclin1, LC3-II/LC3-I and LAMP-2, in podocytes in a time- and dose-dependent manner. We also found that PA increased LAMP-2 expression and colocalization with LC3 in podocytes stably expressing GFP-LC3. These results showed that PA induced autophagy. To investigate the effect of PA on autophagic flux in podocytes, an autophagy flux assay was conducted. We found that chloroquine significantly increased the accumulation of GFP-LC3-positive autophagosomes and the expression of LC3-II/LC3-I and LAMP-2 in podocytes and that 3-MA suppressed the PA-induced formation of GFP-LC3 puncta and expression of LC3. Our data indirectly confirms that PA increases autophagic flux at early stages. Since Beclin1 is mainly involved in the initiation of autophagy, blocking autophagosomal degradation with the lysosomal inhibitor chloroquine did not increase the expression of Beclin1 in podocytes induced by PA.

It has been shown that ROS are essential for autophagy^34^,^35^. In our study, we found that palmitic acid promoted the generation of ROS in podocytes and that the antioxidant reagent NAC significantly suppressed the PA-induced autophagosome formation and autophagy-related proteins expression, suggesting that palmitic acid induces podocyte autophagy through ROS generation.

ROS are products of normal metabolism and xenobiotic exposure. Low levels of ROS function as “redox messengers”, which are important for cell signaling and homeostasis; however, excess ROS could lead to cell damage and apoptosis^36^. Mitochondria are both the major source of intracellular ROS production and targets of ROS^37^. In the present study, we found that palmitate induced intracellular lipid accumulation and a decrease in mitochondrial membrane potential (ΔΨm) in podocytes, which indicated that the abnormal accumulation of lipids may induce mitochondrial injury in podocytes. Furthermore, we confirmed that PA increased podocyte apoptosis in a dose-dependent manner, the expression of cleaved-caspase3 and ROS production, and the antioxidant reagent NAC ameliorated PA-induced apoptosis. Taken together, these data provide evidence that ROS plays a key role in mediating PA-induced autophagy and apoptosis in podocytes.

Autophagy is a crucial mechanism that plays an essential role in cell survival under certain stress conditions such as nutrient deprivation. However, under other conditions, uncontrolled massive autophagy may promote cell death, which is described as type II programmed cell death (PCD)^32^,^33^. Mounting evidence shows that autophagy could play a protective role in kidney maintenance and diseases; furthermore, autophagy could also play a negative role in renal diseases such as obstructive uropathy^38^. Therefore, further research is needed to elucidate the exact role of autophagy in podocyte injury induced by lipids. In the present study, we found that the inhibition of autophagy by CQ promoted palmitic acid-induced apoptosis accompanied by the increased generation of ROS in podocytes and that rapamycin-induced autophagy remarkably suppressed palmitic acid-induced ROS generation and apoptosis in podocytes, indicating that autophagy serves as a protective mechanism against apoptosis in podocytes under stress, potentially through the amelioration of excessive ROS production. However, the exact mechanism by which autophagy suppresses excessive ROS production remains to be elucidated. In fact, when mitochondria are damaged, high levels of ROS are released into the cytoplasm, thus damaging other organelles and activating autophagy to remove non-functional proteins and organelles. If this is not sufficient, cell death will be induced^37^. However, the exact mechanism by which autophagy protects podocytes under high lipid conditions is still uncertain and is the subject of ongoing investigations.

**Figure 8:**
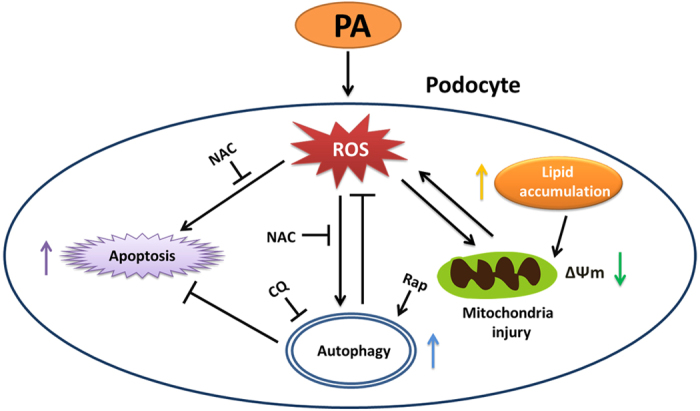
Hypothetical mechanism for palmitic acid-induced autophagy and apoptosis in podocytes. PA exposure induces an abnormal accumulation of lipids, which may induce mitochondrial injury. Mitochondria are both the major source of intracellular ROS production and targets of ROS, and mitochondrial damage can lead to excessive ROS generation, which can induce autophagy and apoptosis. On the other hand, autophagy serves as a protective mechanism through an unknown pathway, reducing excessive ROS production and protecting against palmitic acid-induced apoptosis in podocytes.

In conclusion, we have shown that palmitic acid induced lipid accumulation, mitochondrial injury, the activation of autophagy, and apoptosis in podocytes. PA-induced autophagy and apoptosis in podocytes were associated with excessive ROS production, which may be derived from damaged mitochondria. In addition, autophagy served as a protective mechanism against excessive ROS production and apoptosis in podocytes. The hypothetical cellular and molecular events regulating palmitic acid-induced autophagy and apoptosis in podocytes are summarized in Fig. 8. Further studies are required to determine which organelles participate in autophagy and how it is regulated in podocytes under high lipid conditions, with the aim of developing a therapeutic approach for patients with kidney diseases accompanied by hyperlipidemia, such as DN patients.

## Materials and Methods

### Podocyte culture

The conditionally immortalized mouse podocyte cell line (MPC5) was a kind gift from Dr Ruan at the Centre for Nephrology, Royal Free and University College Medical School, London, United Kingdom. The cells were grown on type I collagen-coated dishes in RPMI-1640 medium supplemented with 10% fetal bovine serum (FBS, Gibco), 1000 U/L penicillin, 100ug/mL streptomycin and 10 U/ml recombinant mouse γ-interferon in an atmosphere containing 5% CO_2_ at 33 °C. To induce podocyte differentiation, the cells were cultured at 37 °C without interferon-γ for 14 days, and then the following experiments were performed.

### Establishment of MPC5 cells stably expressing green fluorescent protein fused to microtubule-associated protein light chain 3 (GFP-LC3)

As the expression of GFP-LC3 is now widely used to visualize autophagy in cultured cells, we established a stable GFP-LC3-expressing MPC5 cell line by following the steps below. Podocytes were cultured according to the above protocol at 33 °C and infected with GFP-LC3 lentivirus (Genechem, LV-MAP1LC3B, 3905-1) at an MOI of 10 for 48 hours. MPC5 cells stably expressing GFP-LC3 were screened with puromycin at a concentration of 2 ug/mL and analyzed with fluorescent microscopy for infection efficiency.

### Autophagy was evaluated by GFP-LC3 puncta formation

After treatment with 150 μmol/L palmitic acid for 24 hours, stable GFP-LC3-expressing podocytes were washed with PBS, fixed with 4% paraformaldehyde and stained with 4, 6-diamidino-2-phenylindole (DAPI; Invitrogen, USA) for 3 minutes. After three washes with PBS, the cells were visualized under a fluorescence microscope.

### Immunocytochemistry

Stable GFP-LC3-expressing podocytes were seeded in 12-well plates with glass coverslips, and then treated with 150 μmol/L palmitic acid for 24 hours. The cells were fixed with 4% paraformaldehyde for 15 minutes, permeabilized with 0.1% Triton X-100 for 3 minutes and blocked with 5% BSA for 1 hour at room temperature. The cells were incubated with a rabbit anti-LAMP2 antibody (1:200, Abcam) at 4 °C overnight and then washed three times with PBS and incubated with an Alexa Fluor 546 donkey anti-rabbit IgG (1:400, Invitrogen, A10040, USA) for 1.5 hours. After three washes with PBS, the cells were stained with DAPI for 3 minutes and visualized with a fluorescence microscope.

### Evaluation of autophagic vacuoles by monodansylcadaverine (MDC) staining

Podocytes were treated with 150 μmol/L palmitic acid for 24 hours. The cells were then incubated with 0.05 mM MDC (Sigma-Aldrich, USA) at 37 °C for 15 minutes. After incubation, the cells were washed three times with PBS and immediately examined with a fluorescence microscope at an excitation wavelength of 330 nm and emission wavelength of 515 nm.

### Western blot analysis

After treatments, the cells were washed twice with ice-cold PBS and lysed in RIPA lysis buffer (Beyotime, Beijing, China) with protease inhibitors, sonicated for 15 seconds and centrifuged at 12,000 g for 15 minutes at 4 °C. The protein concentrations were determined by a BCA protein assay kit (Beyotime, Beijing, China), and then the protein samples were mixed with loading buffer and heated at 100 °C for 10 minutes. Equal amounts of protein samples were loaded per lane, separated by 10% SDS-PAGE, and transferred onto PVDF membranes (Millipore), which were then blocked with 5% non-fat milk for 3 hours. The PVDF membranes were then probed with different antibodies: rabbit anti-LAMP2 (1:1500, Abcam), rabbit anti-Beclin1 (1:1000, CST), rabbit anti-LC3 (1:1000, CST), rabbit anti-cleaved-caspase3 (1:1000, CST) and mouse anti-β-actin (1:5000, Sungene Biotech). After the primary antibody binding, the membranes were incubated with horseradish peroxidase (HRP)-labeled goat anti-rabbit (1:8000, MultiSciences, GAR007, China) or goat anti-mouse IgG (1:8000, MultiSciences, GAM007, China) at room temperature for 1 hour. The reactive proteins were detected by an ECL chemiluminescence system (GE Healthcare, Piscataway, NJ, USA), the band intensities were quantified with Quantity One software, and the results were normalized to β-actin.

### Measurement of lipid uptake

Lipid uptake was observed by Oil Red O staining and BODIPY lipid probes. To measure the uptake of fatty acid by Oil Red O staining, cells were fixed in 4% formaldehyde for 15 minutes, washed with PBS, and stained with Oil Red O working solution for 30 minutes. After removing the Oil Red O solution, the cells were immediately washed with 60% isopropanol for 5 seconds, and then treated with hematoxylin for 5 minutes. To measure the uptake of fatty acid by BODIPY lipid probes, podocytes were incubated with BODIPY lipid probes (10 μg/ml, BODIPY500/510 C1, C12, Invitrogen, USA) at 37 °C for 1 hour, washed 3 times with PBS and immediately examined under a fluorescence microscope.

### Measurement of reactive oxygen species (ROS) in podocytes

Intracellular ROS generation was detected using the 2′,7′-dichlorofluorescein diacetate (DCFH-DA) fluorescent probe. Pretreated cells in 12-well plates were incubated with DCFH-DA (10 μmol/L) at 37 °C for 30 minutes and then washed with PBS. The cell fluorescence were observed under a fluorescence microscope at an excitation wavelength of 488 nm and emission wavelength of 525 nm.

### Determination of mitochondrial transmembrane potential (∆Ψm)

The mitochondrial membrane potential of podocytes was detected by a mitochondrial membrane potential assay kit (JC-1, Beyotime, China). The kit utilizes 5,5′,6,6′-tetrachloro-1,1′,3,3′-tetraethyl-imidacarbocyanine iodide (JC-1), a cationic dye that accumulates in energized mitochondria, to detect variations in mitochondrial membrane potential. According to the manufacturer’s instructions, podocytes were seeded on a glass coverslip in 12-well culture plates and then treated with 150 μmol/L palmitic acid for 24 hours, washed once with PBS and incubated with 500 uL JC-1 staining working solution for 20 minutes at 37 °C. After three washes with JC-1 staining buffer, fluorescence images were captured using a fluorescence microscope. Carbonyl cyanide 3-chlorophenylhydrazone (CCCP), a powerful mitochondrial uncoupling agent, was used as a positive control.

### Flow cytometry analysis

Apoptosis was determined using the Annexin V-FITC/PI apoptosis assay kit (Sungene Biotech, China). Flow cytometry with a BD FACSVantage SE cytometer was used to detect the apoptosis rate of cells. Annexin V-positive/PI-negative podocytes were considered to indicate the early stages of apoptosis, whereas annexin V-positive/PI-positive podocytes were considered to be late apoptotic or necrotic cells.

### Statistical analysis

The data were expressed as the mean ± SEM. Statistical analyses were performed using Graph Pad Prism (GraphPad software). Differences between 2 groups were analyzed using Student’s t-test, and multiple comparisons were analyzed with a one-way ANOVA. A P-value < 0.05 was defined as statistically significant.

## Additional Information

**How to cite this article**: Jiang, X.- *et al*. Autophagy Protects against Palmitic Acid-Induced Apoptosis in Podocytes in vitro. *Sci. Rep.* **7**, 42764; doi: 10.1038/srep42764 (2017).

**Publisher's note:** Springer Nature remains neutral with regard to jurisdictional claims in published maps and institutional affiliations.
